# Mezcal worm in a bottle: DNA evidence suggests a single moth species

**DOI:** 10.7717/peerj.14948

**Published:** 2023-03-08

**Authors:** Akito Y. Kawahara, Jose I. Martinez, David Plotkin, Amanda Markee, Violet Butterwort, Christian D. Couch, Emmanuel F.A. Toussaint

**Affiliations:** 1 McGuire Center for Lepidoptera and Biodiversity, Florida Museum of Natural History, University of Florida, Gainesville, FL, United States of America; 2 School of Natural Resources and Environment, University of Florida, Gainesville, FL, United States of America; 3 Ontario Forest Research Institute, Ministry of Natural Resources and Forestry, Sault Ste. Marie, Ontario, Canada; 4 Natural History Museum of Geneva, Geneva, Switzerland

**Keywords:** Agave redworm moth, *Comadia redtenbacheri*, Cossidae, Goat moth, Gusano del maguey, Identification, Liquor, Food science, Tequila

## Abstract

Mezcals are distilled Mexican alcoholic beverages consumed by many people across the globe. One of the most popular mezcals is tequila, but there are other forms of mezcal whose production has been part of Mexican culture since the 17th century. It was not until the 1940–50s when the mezcal worm, also known as the “tequila worm”, was placed inside bottles of non-tequila mezcal before distribution. These bottled larvae increased public attention for mezcal, especially in Asia, Europe, and the United States. Despite these larvae gaining global interest, their identity has largely remained uncertain other than that they are larvae of one of three distantly related holometabolous insects. We sequenced the COI gene from larvae in different kinds of commercially available mezcals. All larval DNA that amplified was identified as the agave redworm moth, *Comadia redtenbacheri.* Those that did not amplify were also confirmed morphologically to be the larva of this species.

## Introduction

Mezcal is a traditional, Mexican distilled alcoholic beverage made from the plant genus *Agave* Linnaeus. While tequila is a specific, popular type of mezcal made from blue agave (*A. tequilana* F.A.C. Weber), mezcal can be distilled from 30 of the 159 species of Mexican agaves (McEvoy, 2018). Mezcal production begins with the heart of the plant being boiled for several days in underground pit ovens, allowing it to obtain its intense and distinctive smoky flavor. Cooked agave hearts are mashed and left to ferment in large barrels containing water and are distilled twice in pots and left to age in barrels between one month to several years (McEvoy, 2018). While more than 70% of mezcals are distilled and bottled in Oaxaca, Mexico (Barbezat, 2020), mezcal is now exported throughout the world with growing global demand (McEvoy, 2018). However, this traditional beverage is threatened by a shortage and a rise in prices of raw materials as the demand for tequila rises (Bautista, Orozco-Cirilo & Terán-Melchor, 2015). The increased difficulty in turning a profit from mezcal is likely to discourage local distillers, putting the entire tradition at risk (Espinosa-Meza, Rivera-González & Maldonado-Ángeles, 2017).

Although mezcal has been part of Mexican culture since the seventeenth century (Zizumbo-Villarreal & Golunga-GarciaMarin, 2007), distillers did not start placing a “mezcal worm” inside the bottle until the 1940–50s (Greene, 2017). Mexican entrepreneur Jacobo Lozano Paez is thought to have been the first “maestro mezcalero” or “mezcal master” to place larvae in bottles as a marketing strategy, to enhance the flavor and color of the drink (Greene, 2017). Notably, none of these mezcal brands are tequila, as authentic tequila never includes a worm (Téllez & Estrada, 2012). There are still many mezcal brands that refrain from participating in the twentieth-century novelty of including larvae or other ingredients (such as fruits and scorpions; Carrillo-Trueba, 2007). Some conservative mezcal producers claim that superfluous inclusion of larvae only lowers the quality of the final product (Stewart, 2013; McEvoy, 2018).

**Figure 1:**
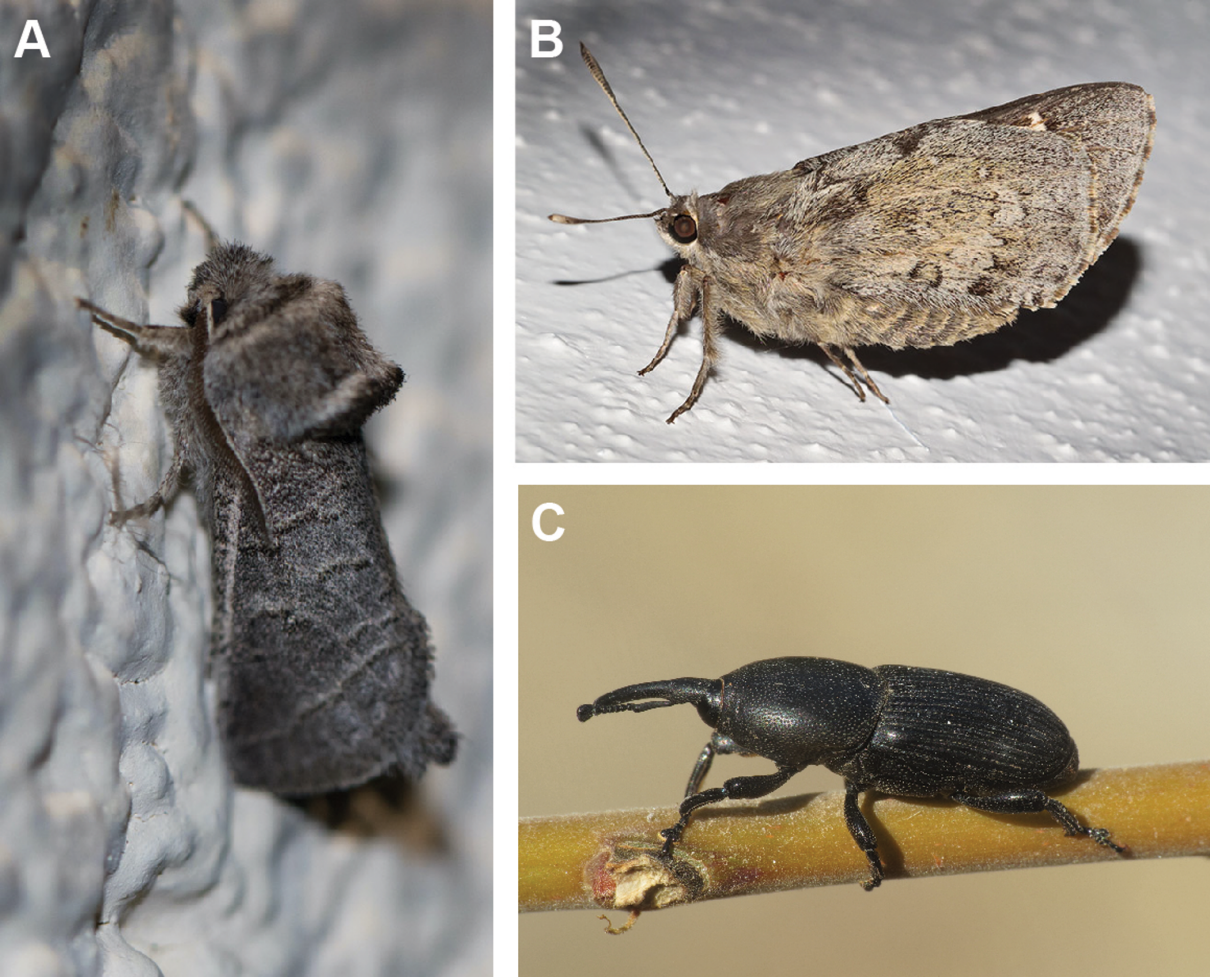
Adults of three insects species presumed to be the mezcal worm. (A) *Comadia redtenbacheri* (Cossidae), (B) *Aegiale hesperiaris* (Hesperiidae), (C) *Scyphophorus acupunctatus* (Curculionidae). Photo credits (from (A–C), respectively): Mark Rosenstein, Big Bend, Brewster, Texas, USA; Ricardo Arredondo T., Coeneo, Michoacan, México; Simon Oliver, Yegen, Granada, Spain.

It is well known that the mezcal worm is the larva of a holometabolous insect (Finch & Zarazaga, 2007; Molina-Vega et al., 2021). However, there is conflicting information on the identity of the larva of the species that is in mezcals. Literature suggests that the worm is one of three different insects in two different insect orders (Finch & Zarazaga, 2007; Molina-Vega et al., 2021; Fig. 1). The larva is usually either a white or red “maguey worm” (maguey means agave in Spanish) (Van Huis, 2013; Molina-Vega et al., 2021). White maguey worms are thought to be the larva of the agave snout weevil (Coleoptera: Curculionidae: *Scyphorphorus acupunctatus* Gyllenhaal) (Lacy, 1988; Finch & Zarazaga, 2007) or the Tequila giant skipper, *Aegiale hesperiaris* (Walker), family Hesperiidae (Lepidoptera). The weevil is known as “picudo del agave,” a major pest of agave and yucca in Mexico (Cuervo-Parra et al., 2019). A gravid female weevil punctures the lower part of the agave plant, including the trunk and external roots. Eggs are deposited singly or in clusters at these punctures after the onset of tissue decay. Eggs hatch after ∼5 days and larvae burrow into the agave tissue and require about 50 to 90 days to mature to pupation; pupation typically lasts 11 days to 2 weeks. The weevil is known to introduce bacteria and microorganisms that can further harm the plant (Cuervo-Parra et al., 2019). The larva of the Tequila giant skipper is also known as “meocuiles” or “meocuilines” from the Nahuatl words metl = maguey or agave and ocuilin = worm. This butterfly larva feeds on leaves of *Agave salmiana* Otto ex Salm-Dyck, *Ag. mapisaga* Trel. and *Ag. tequilana* F.A.C. Weber (García-Rivas, 1991; Ramos-Elorduy et al., 2011; Molina-Vega et al., 2021). Adult females of *Ae. hesperiaris* deposit up to 14 eggs, usually in clusters, near the base of agave leaves in autumn. Eggs hatch after 15–40 days and larvae enter the plant by cutting an opening on the underside of the leaf (Jaimes-Rodríguez et al., 2020; Vargas-Zuñiga et al., 2019). Local collectors harvest wild larvae between May and July by identifying infected agave plants and extracting the larva using a hook. *Aegiale hesperiaris* is highly prized because of its exquisite taste and may be locally threatened due to overcollection and habitat loss (Ramos-Elorduy, 2006).

Red maguey worms are called “gusano rojo de maguey,” “chinicuil,” or “chilocuil,” which comes from the Nahuatl words chilo = pepper and ocuilin = worm; hence, the “chili worm” (Molina-Vega et al., 2021). Red maguey worms are thought to be the caterpillar of the Agave redworm moth (Lepidoptera: Cossidae: *Comadia redtenbacheri* (Hammerschmidt, 1848)) (Molina-Vega et al., 2021). The larva of *C. redtenbacheri* feeds on *A. americana, A. atrovirens*, *A. mapisaga,* or *A. salmiana* (Molina-Vega et al., 2021). A female can lay approximately 120 eggs which hatch in approximately one month, and larvae feed on the roots and stems (Camacho et al., 2003; Llanderal-Cázares et al., 2007). Larvae form colonies of 40–60 individuals at the base of fleshy leaves along the agave stem (Molina-Vega et al., 2021). Like most Cossidae larvae, *Comadia redtenbacheri* are red, but unlike other cossid species, *C. redtenbacheri* larvae develop in agaves instead of in tree trunks, roots, crowns, stems, or branches (Vergara et al., 2012; Castro-Torres & Llanderal-Cázares, 2016). Because these moths aggregate in large numbers within the plant, when this moth is harvested, the agave dies (Molina-Vega et al., 2021).

Local worm collectors or “gusaneros” harvest wild larvae of red and white maguey worms by hand between May and September, when they are most abundant. Gusaneros identify infected agave plants and extract larvae using a metal hook or an agave spine, avoiding as much damage to plants as possible (Ramos-Elorduy, 2006; Miranda-Román et al., 2011). Larvae are collected in the field from wild populations and are not industrially produced (Molina-Vega et al., 2021). Because maguey worms are highly prized for their exquisite taste, they may be locally threatened due to overcollection and habitat loss (Ramos-Elorduy, 2006; Llanderal-Cázares et al., 2007).

Red and white maguey worms are rich in protein (35–65% dry basis), fat (13–33%), vitamins (B_1_, B_2_, B_6_, C, D, E, K), contain sodium, potassium, iron, zinc, calcium, copper, phosphorus, magnesium, and manganese (Rumpold & Schlüter, 2013; Schluter et al., 2017; de Castro et al., 2018; Kim et al., 2019). Their high sodium and potassium content can help lower blood pressure and prevent arterial hypertension, cardiopathies, and strokes in consumers when compared to foods like beef, fish, beans, peas, and potatoes (Molina-Vega et al., 2021).

Although these larvae are popular in Mexican cuisine because of their unique flavor and high protein and fat content, there is still no consensus on which insect species is found in modern mezcal bottles. Are people consuming larvae of the skipper butterfly *A. hesperiaris,* or the larva of the moth *Comadia redtenbacheri*, the latter which is thought to be declining in numbers in recent years? Or is the worm the larva of a weevil, or another unidentified insect species? Here we determine the identity of these larvae by conducting a DNA-based identification analysis of larvae inside 21 commercially available mezcals.

## Materials & Methods

### Sample collection and data recording

**Figure 2:**
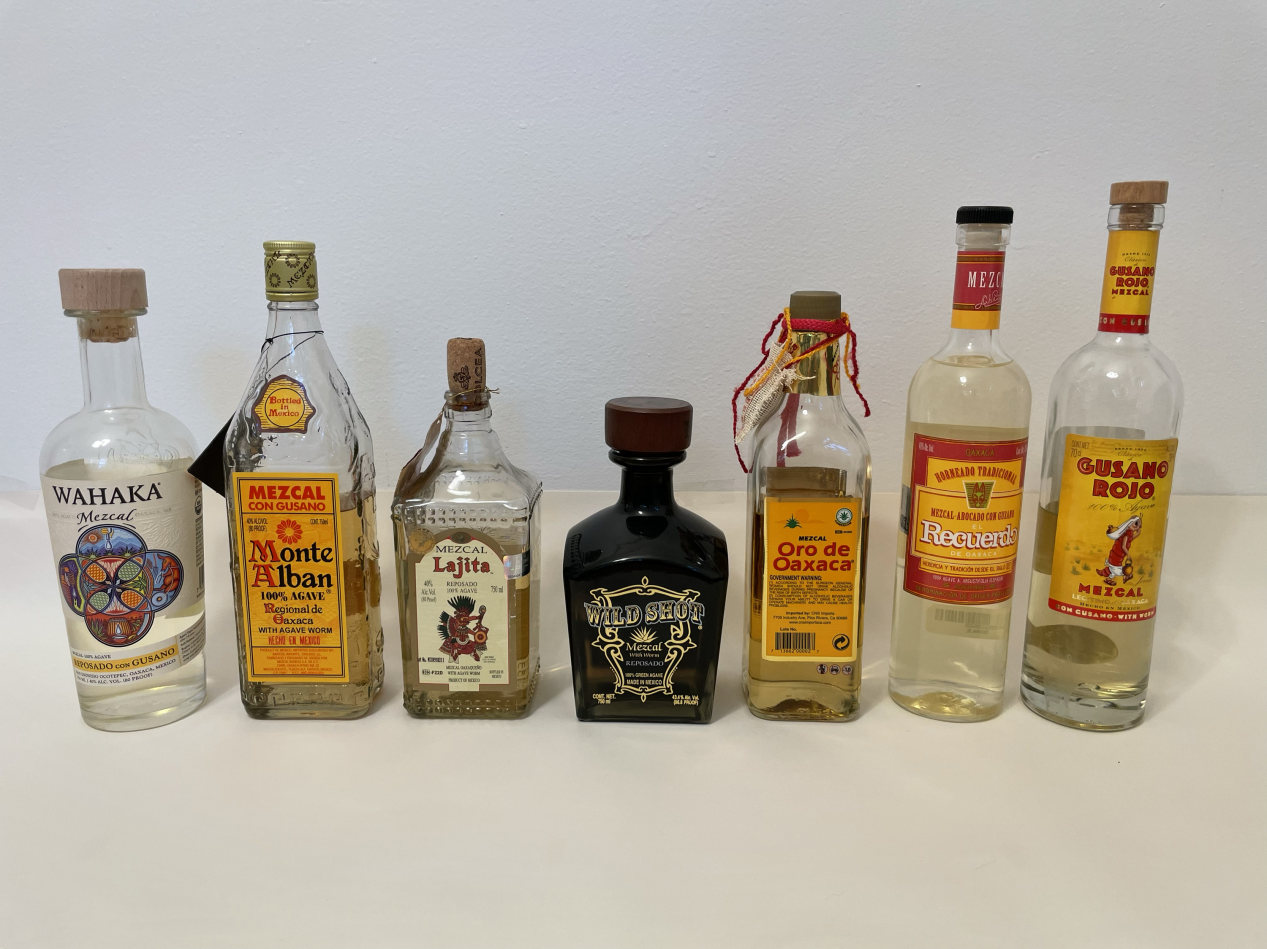
Different kinds of mezcals tested for the identity of “mezcal worms.”. Worms have been removed from bottles in the image. Photo by Akito Y. Kawahara.

**Figure 3:**
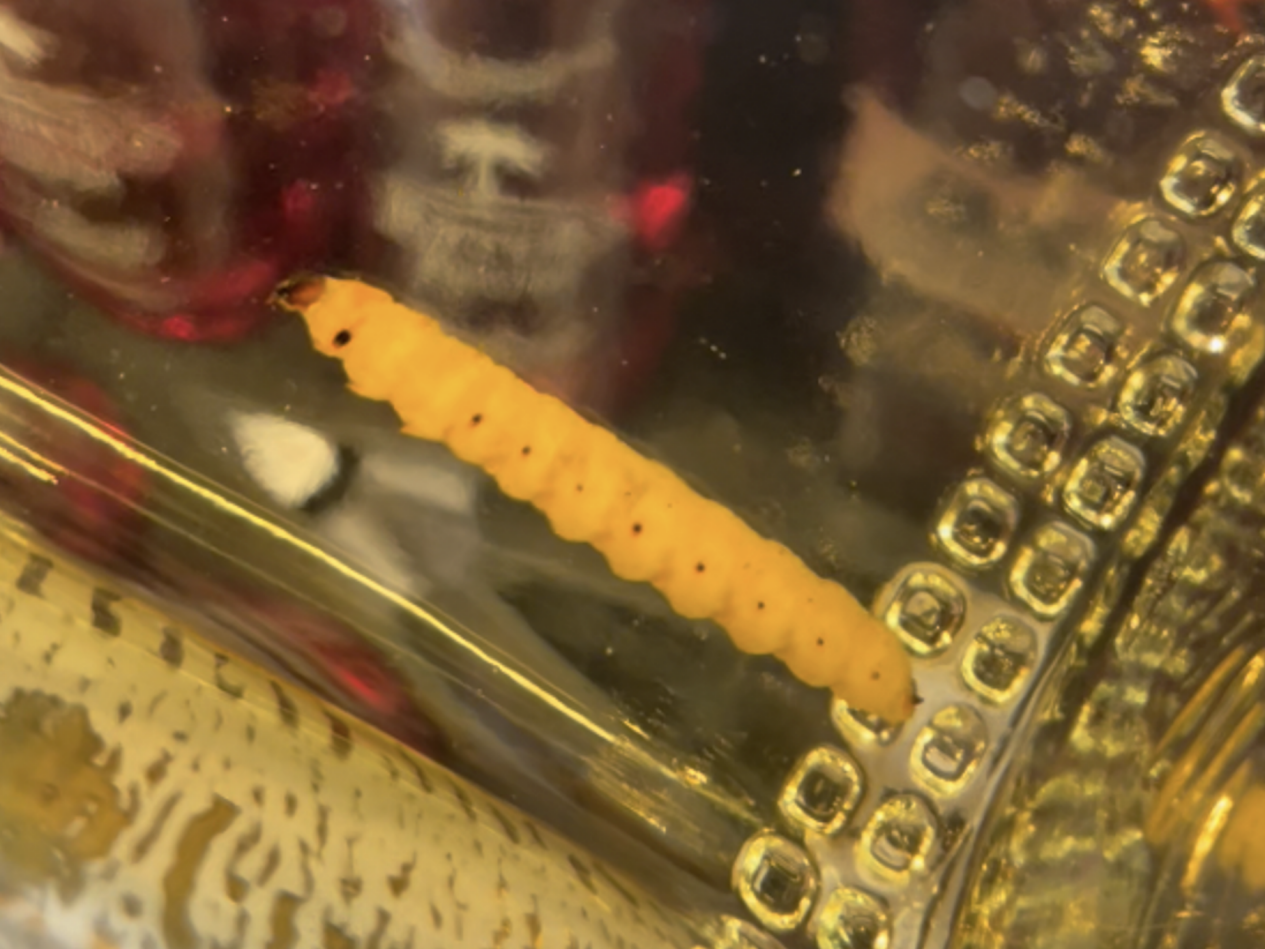
Closeup image showing a worm inside a bottle of “Lajita Reposado” mezcal. Photo by Akito Y. Kawahara.

Specimens were obtained from mezcal bottles that were purchased between 2018 and 2022 (Fig. 2). We attempted to obtain as many mezcals as possible that contain larvae, both from North American distributors, and from distilleries that we visited in Oaxaca, Mexico, in November 2022. The larva (Fig. 3) was removed from the bottle by using a five cm diameter round metal sifter, which was placed over a 2-cup mason jar. The mezcal and worm were poured through the sifter, and the larva retrieved. Each larva was photographed using a Canon EOS 7D camera with a Canon EF-S 60 mm f/2.8 USM Macro Lens, from the dorsal and lateral sides and later transferred to a 25 ml polypropylene centrifuge tube containing 95% undenatured ethanol at the McGuire Center for Lepidoptera and Biodiversity (MGCL), Florida Museum of Natural History, University of Florida, Gainesville, FL, USA. Data for each specimen (*i.e.,* from which bottle the specimen was taken) was recorded. All tissues are deposited in tubes containing 95% ethanol, stored in the −80 °C freezer collection at the MGCL.

### DNA extraction and sequencing

Specimens were removed from the −80 °C freezer and a small 0.5–1 mg piece of tissue from the cross-section of the thorax was dissected. Tissues were individually placed in wells of a 96-well plate and sent either to the Canadian Center for DNA Barcoding (CCDB) in Guelph, Canada, or to the Smithsonian Institution, Washington, D.C. for DNA extraction and sequencing. In both cases, specimens were sequenced with Sanger sequencing using the cytochrome c oxidase subunit 1 (COI) LCO-HCO primers (Folmer et al., 1994). Samples that failed for sequencing were re-extracted using the The Extract-N-Amp™ DNA extraction kit (Sigma Aldrich, St. Louis, MO, USA) or the OmniPrep DNA extraction kit (G-Biosciences, St. Louis, MO, USA) at the MGCL. For extractions that used the Extract-N-Amp kit, we largely followed the manufacturer’s protocol, summarized here: 40 µL of Extract-N-Amp extraction solution was added to each 0.2 mL PCR tube that contained a small piece of larval thoracic tissue. The tissue was gently macerated and incubated on a thermocycler at 96 °C for 30 min. Once finished, an equal volume to the extraction solution of 3% BSA was added, and the mixture was vortexed for 15 s before being centrifuged for 15 s. Thirty microliters of supernatant was pipetted into a new 1.5 mL Eppendorf tube and the stock used for PCR.

For samples extracted using the OmniPrep DNA extraction kit, we used the following protocol, slightly modified from the manufacturer’s guidelines: A small piece of larval thoracic tissue was placed in a clean microcentrifuge tube, before 180 µL of OmniPrep genomic lysis buffer and 20 µL of OmniPrep Proteinase K were added to the same tube. After macerating the tissue in this solution, the tube was vortexed for 15 s before being incubated at 56 °C overnight. Afterwords, 100 µL of chloroform was added and the tube contents vortexed for 15 s before being spun on a centrifuge for 10 min at 14,000 RCF. The supernatant was transferred to a new tube and 25 µL OmniPREP DNA stripping solution was added to the tube before the tube was vortexed for 15 s. Tubes and their contents were incubated at 56 °C for 10 min before being cooled to room temperature and 50 µL of OmniPrep precipitation solution and 3 µL OmniPrep mussel glycogen was added. Samples were vortexed for 15 s before being spun on a centrifuge for 20 min at 14,000 RCF. The supernatant was transferred to a new Eppendorf tube and 250 µL of cold isopropanol was added before being incubated at −20 °C for 30 min. The sample was centrifuged for 10 min at 14,000 RCF and 350 µL of cold 80% ethanol was added. Samples were centrifuged for another 10 min at 14,000 RCF and the ethanol discarded. The tube was kept open at room temperature to allow ethanol evaporation. Once sample pellets were dry, 50 µL of OmniPrep TE buffer and 0.5 µL of RNAse were added. We attempted to sequence other genes in addition to COI, but those did not amplify, likely due to sample degradation.

### Sequence assembly and species identification

Forward and reverse COI sequences were aligned and assembled using Geneious 9.1.3 (http://www.geneious.com). Sequence ends were trimmed to exclude primer regions. Additional COI sequences of all available *Comadia* species and two cossid outgroups (*Acossus populi* Walker and *Hypopta palmata* Barnes and McDunnough) were downloaded from the Barcode of Life Data System V4 (https://www.boldsystems.org/) and GenBank databases (http://www.ncbi.nlm.nih.gov/Genbank). Specifically, we downloaded fourteen sequences, and their associated data are listed in File S1.

Of the eleven described species of *Comadia* (Brown, 1975), only two (*C. redtenbacheri* and *C. henrici* Grote) have COI data available on GenBank, as of January 2023. We compared our sequences visually in Geneious and then calculated similarity scores (*e*-value) to published sequences with nucleotide BLAST (https://blast.ncbi.nlm.nih.gov/Blast.cgi). We also calculated pairwise distances between every sample in our dataset to examine the percent sequence difference. Pairwise distances were calculated in PAUP* version 4.0a169 (Swofford, 2003) with default settings and exported with the “SaveDist” command.

We also reconstructed a COI tree using the maximum-likelihood (ML) optimality criterion in the software IQ-TREE *v.* 2.1.0 (Nguyen et al., 2015; Minh et al., 2020), using default settings with 1,000 tree searches. Branch support was estimated with 1,000 replicates of the Shimodaira-Hasegawa approximate likelihood ratio test (SH-aLRT) and 1,000 replicates of ultrafast bootstraps (UFBoot2; Hoang et al., 2018). We used FigTree v1.4.4 (Rambaut et al., 2018) for tree visualization. Nodes with values of SH-aLRT ≥ 80 and UFBoot2 ≥ 95 were considered to have strong support.

Finally, the second and third authors of this study compared the morphology of larvae in bottles (File S2) to the known morphology of all three putative mezcal worm species to verify our molecular identifications. Because a dichotomous key does not exist for larvae of any of the agave-feeding insects, we could only determine if larvae matched published morphological descriptions of each species. We used five morphological features that are distinct to each species (Table 1) and confirmed our identifications with molecular data when possible.

**Table 1:** Morphological comparison of the larva of three insect species presumed to be the mezcal worm.

	**Head capsule**	**Legs**	**Prolegs**	**Spine on A10**	**Long appendage on A10**	**Reference**
*A. hesperiaris*	Small, angular	Present	Present	Absent	Absent	Jaimes-Rodríguez et al. (2020)
*C. redtenbacheri*	Large, rounded	Present, reduced	Present, reduced	Present	Absent	Castro-Torres & Llanderal-Cázares (2016)
*S*. *acupunctatus*	Large, rounded	Absent	Absent	Absent	Present	Cuervo-Parra et al. (2019)

## Results and Discussion

**Figure 4:**
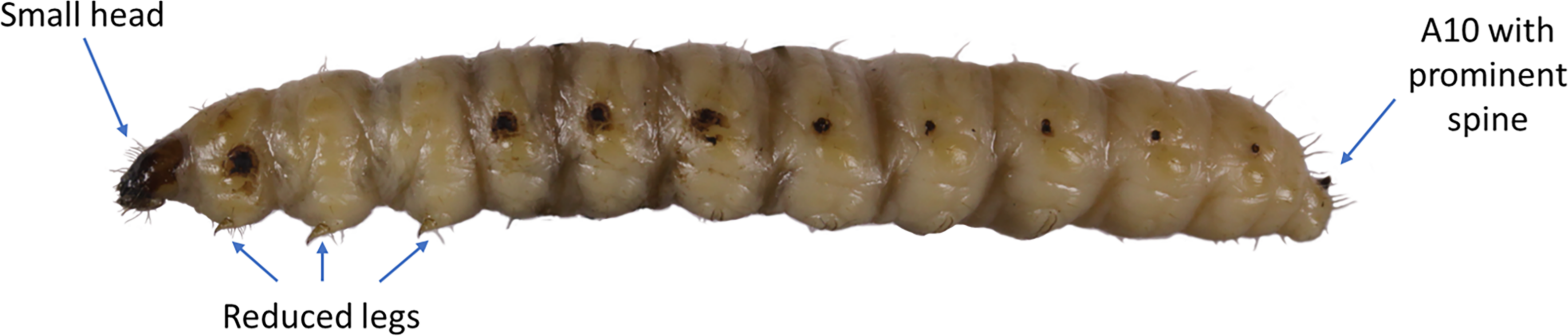
Lateral view of a *Comadia redtenbacheri* larva showing three key features useful to distinguish it from other mezcal worms. Photo by Jose I. Martinez.

We first examined larval morphology. All larvae appeared superficially very similar, with a distinct head capsule and prolegs that are characteristic of lepidopteran larvae. Some specimens were white, others were pinkish red. For samples that had visible diagnostic morphological features, all had a small angular head capsule, reduced legs and prolegs, an upcurved prominent spine on A10, and lacked a pair of long appendages on A10 (Fig. 4). Although some larvae were damaged or missing body parts that prevented definitive identifications, those that retained diagnostic features matched the description of *C. redtenbacheri.*

**Figure 5:**
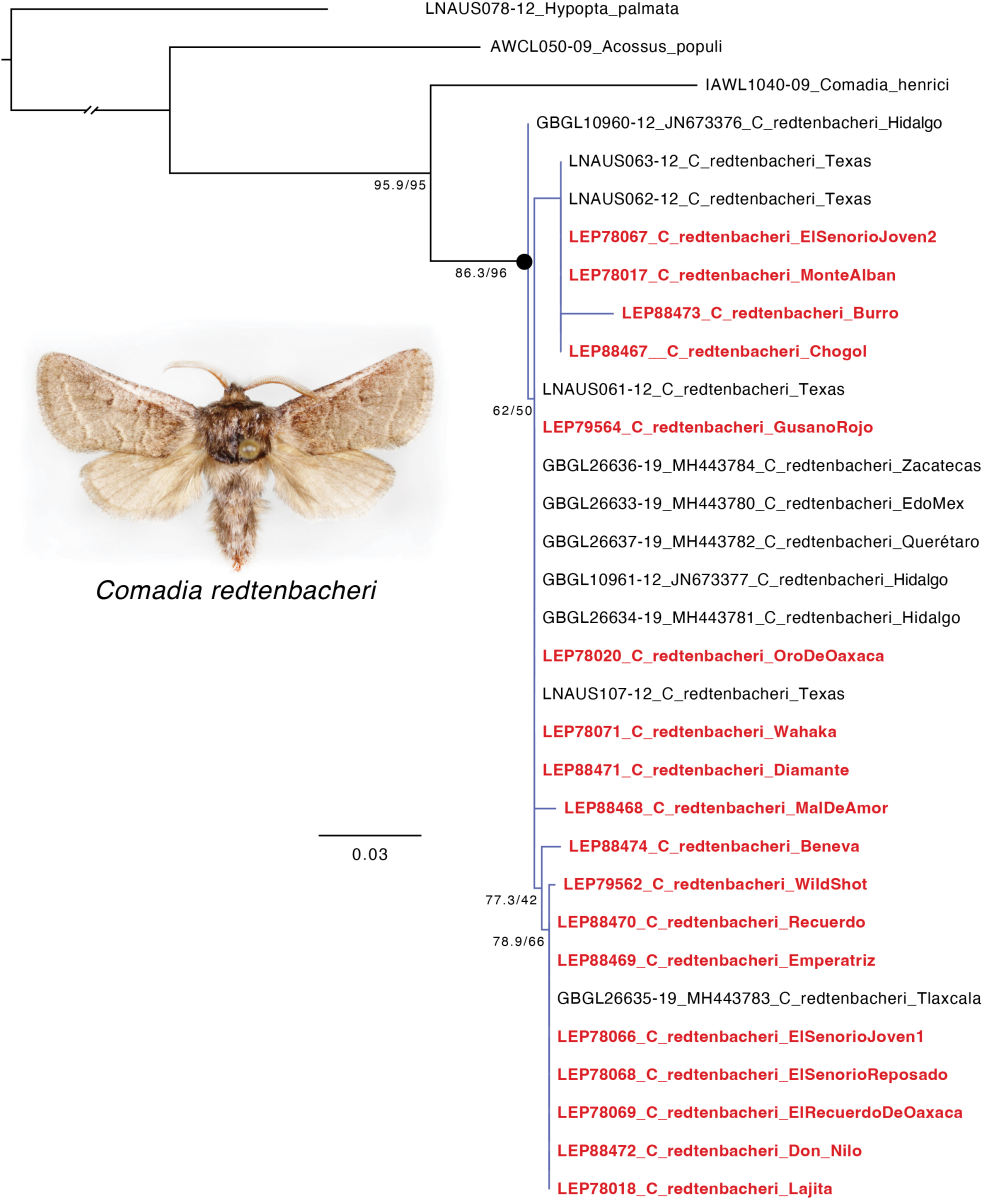
Maximum likelihood tree showing the placement of the 18 successfully sequenced mezcal worms (in bold and red) in relationship to publicly available COI sequences of *Comadia redtenbacheri* (Hammerschmidt) and related Cossidae species. Scale bar = number of substitutions/site. Photo by Jose I. Martinez.

Of the 21 larvae subjected to DNA extraction, 18 yielded DNA sequences (File S3) that were suitable for analysis (the three larvae that failed were identified as *C. redtenbacheri* based on morphology). The 18 sequences had >99.39% hit (*e*-value = 0.0) and varied by <2.5% similarity to publicly available COI sequences of *C. redtenbacheri* for the sequences with known locality information (Fig. 5, Table 2, File S4). The COI gene tree had all species presumed to be *C. redtenbacheri* grouping together as monophyletic and this clade had strong support (SH-aLRT = 86.3; UFBoot2 = 96), but there was no notable clustering of specimens based on geography for the sequences with known locality information (Fig. 5, File S5). Based on sequence similarity to *C. redtenbacheri* in existing databases and placement on the COI tree, we identified all 18 specimens that we sequenced as being *C. redtenbacheri.*

Our result was somewhat unexpected because there are historically about 63 species of larvae or “worms” that are consumed in Mexico, including the Tequila giant skipper (*A. hesperiaris*) which, given its name, implies that it is included in tequila and other mezcals (Ramos-Elorduy et al., 2011). Anecdotal reports of white worms in mezcal bottles are likely due to red agave worms losing their color when stored in alcohol, resulting in a yellowish-white or white appearance (Millán-Mercado et al., 2016). Furthermore, the low abundance of wild *A. hesperiaris* populations, combined with their high price in the food market (roughly US$250.00 per kilogram), makes it unlikely that a mezcal distiller would include *A. hesperiaris* larvae in mezcal bottles (Ramos-Elorduy, 2006; Espinosa-Meza, Rivera-González & Maldonado-Ángeles, 2017).

Gusaneros have continued the century-old tradition of collecting mezcal worms which predates the expanded mezcal production of the late twentieth and early twenty-first centuries (García-Rivas, 1991). Local collectors can differentiate edible larvae by morphology, life history, and/or host plant association (Ramos-Elorduy, 2006). Therefore, it is possible that the name “Tequila giant skipper” was misleadingly applied to the butterfly simply because its larva were collected from blue agave (*A. tequilana*), the plant used to make tequila (Molina-Vega et al., 2021). The same is likely true for the weevil *S. acupunctatus*, which feeds on agaves, just like *C. redtenbacheri* (Molina-Vega et al., 2021). Furthermore, of the eleven described *Comadia* species, only *C. redtenbacheri* feeds on agave (Cárdenas-Aquino et al., 2018), and it is also the only species of *Comadia* known from Mexico (Brown, 1975). For these reasons, it is unlikely that another species of *Comadia* is included in mezcal bottles. However, it should be noted that our results are based on a sample size of 18 mezcals that contain larvae. While we believe our sampling is a solid representation of the breadth of mezcals that contain larvae, it is possible that additional brands and varieties that we could not sample may contain larvae of other insect species.

It remains unknown why three of the 21 larvae did not yield DNA. We tried different extraction protocols and there was no correlation between alcohol percentage and DNA extraction success (Table 2). While it is thought that mezcals originally had live larvae placed in bottles, many distilleries nowadays toast larvae before placing them in bottles for hygienic purposes (Millán-Mercado et al., 2016). Cooking larvae prior to bottling could significantly fragment the DNA of these three larvae. Alternatively, it may be that these larvae had their DNA degraded from other factors such as warm storage conditions, UV exposure, or elements in the liquor.

**Table 2:** Different mezcal worms tested in this study, including the brand, alcohol percentage, and year and month of the mezcal bottle obtained. A statistical comparison of DNA sequences obtained with those available online via BLAST is also included. “Alc% “ and “Vol.” refer to the alcohol percentage and volume of the bottle from which the sample was obtained. “Date” = the month and year which the bottle was obtained; “e-val = *e*-value; “Top hit” = the accession code for the *C. redtenbacheri* GenBank sequence that has the closest hit by % identity (% ID); “Omni-P” = Omni-Prep extraction kit; “Host plant” = agave species from which the larval was likely taken from. Three samples, indicated by asterisks, were extracted for DNA but failed for sequencing.

**Name**	**Company**	**Alc%**	**Vol.**	**Date**	**Unique ID**	**e-val**	**Top hit**	**% ID**	**Seq. L**	**Extraction**	**Host plant**	**GenBank**
Chogol Mezcal/ Organico Artesanal	Chogol Mezcal/ ITSCo	40	375 ml	12-2022	LEP88467	0	JN673377.1	98.77	692 bp	Ex-inAmp,Omni-P	*A. angustifolia*	OQ290806
Don Nilo	Don Nilo	38	150 ml	12-2022	LEP88472	0	JN673377.1	99.69	691 bp	Ex-inAmp,Omni-P	*A. angustifolia*	OQ290808
El Recuerdo de Oaxaca Mezcal	Recuerdo de Oaxaca	38	50 ml	10-2018	LEP78069	0	JN673377.1	99.54	679 bp	Omni-P	*A. angustifolia*	OP654754
El Señorio (Joven con gusano) #1	Bugarin Exportaciones	38	50 ml	10-2018	LEP78066	0	JN673377.1	99.54	678 bp	Omni-P	*A. angustifolia*	OP654756
El Señorio (Joven con gusano) #2	Bugarin Exportaciones	38	50 ml	10-2018	LEP78067	0	JN673377.1	100	597 bp	Omni-P	*A. angustifolia*	OP654761
El Señorio (Reposado con gusano)	Bugarin Exportaciones	38	50 ml	10-2018	LEP78068	0	JN673377.1	99.54	679 bp	Omni-P	*A. angustifolia*	OP654753
Emperatriz del Mezcal Artesanal/ Abocado con Gusano	Emperatriz del Mezcal	36.6	375 ml	12-2022	LEP88469	0	JN673377.1	98.89	693 bp	Ex-inAmp,Omni-P	*A. angustifolia*	OQ290809
Gusano Rojo Mezcal	Gusano Rojo	38	700 ml	5-2019	LEP79564	0	JN673377.1	100	682 bp	Omni-P	*A. angustifolia*	OP654757
Huipil Mezcal con Gusano y Caramelo*	Destiladora de Mezcal Mezcalero	37	750 ml	4-2020	LEP34004	N/A	N/A	N/A	N/A	Ex-inAmp,Omni-P	*A. angustifolia*	N/A
Lajita Mezcal	Licores Veracruz	40	750 ml	5-2019	LEP78018	0	JN673377.1	99.54	677 bp	Omni-P	*A. angustifolia*	OP654755
La Penca Mezcal (w/worm)*	Vinicola del Altiplano	40	50 ml	5-2019	LEP79563	N/A	N/A	N/A	N/A	Ex-inAmp,Omni-P	*A. salmiana*	N/A
Mal de Amor/Abocado con Gusano	Palenque Mal de Amor	45	750 ml	12-2022	LEP88468	0	JN673377.1	98.17	692 bp	Ex-inAmp,Omni-P	*A. angustifolia*	OQ290804
Mezcal Beneva	Mezcal Beneva, S.A. de C.V.	38	50 ml	12-2022	LEP88474	0	JN673377.1	98.24	693 bp	Ex-inAmp,Omni-P	*A. angustifolia*	OQ290805
Mezcal Burro/Espadin	Mezcal Burro	48	500 ml	12-2022	LEP88473	0	JN673377.1	96.45	702 bp	Ex-inAmp,Omni-P	*A. angustifolia*	OQ290803
Mezcal Diamante Oaxaqueño	Mezcal Diamante Oaxaqueño	40	50 ml	12-2022	LEP88471	0	JN673377.1	99.05	699 bp	Ex-inAmp,Omni-P	*A. angustifolia*	OQ290807
Monte Alban Mezcal Reposado	Sazerac Company	40	750 ml	5-2019	LEP78017	0	JN673377.1	100	678 bp	Omni-P	*A. angustifolia*	OP654759
Oro de Oaxaca Mezcal w/ Agave Worm	Licorera Oaxaqueña	40	750 ml	5-2019	LEP78020	0	JN673377.1	100	678 bp	Omni-P	*A. angustifolia*	OP654760
Recuerdo de Oaxaca	El Manantial de Matatlan	40	150 ml	12-2022	LEP88470	0	JN673377.1	98.89	695 bp	Ex-inAmp,Omni-P	*A. angustifolia*	OQ290810
Wahaka Mezcal	Wahaka	40	750 ml	5-2019	LEP78071	0	JN673377.1	100	624 bp	Omni-P	*A. angustifolia*	OP654758
Wild Shot, Reposado*	Envasadora La Perla	43.4	750 ml	5-2019	LEP78019	N/A	N/A	N/A	N/A	Ex-inAmp,Omni-P	*A. salmiana*	N/A
Wild Shot, Silver	Envasadora La Perla	43.4	750 ml	8-2019	LEP79562	0	JN673377.1	99.39	683 bp	Omni-P	*A. salmiana*	OP654752

Our finding that all larvae are a single moth species affirms the importance of *C. redtenbacheri* for the mezcal industry. Larvae of *C. redtenbacheri* are one of the most popular edible insects in Mexico (Miranda-Román et al., 2011), and adding them to mezcal bottles brings about the unique color and flavor of the liquor (Greene, 2017). Adding larvae to Mexican beverages and foods (salts, garnishes, powders, *etc*.) is driven by health benefits and by beliefs that these larvae contain aphrodisiac properties (Contreras-Frias, 2013). This trend is resulting in greater demand that is applying pressure to local larval populations (McEvoy, 2018; Molina-Vega et al., 2021). Opportunities for greater income have led some locals to turn to gathering larvae to increase their income (Molina-Vega et al., 2021). Unfortunately, wild-caught larvae are becoming less common, and gatherers are having to travel further to find them (Cisneros, 1988).

In response to the declining number of mezcal larvae, researchers have begun to develop methods to cultivate these larvae in captivity (Molina-Vega et al., 2021). The optimal condition for captive breeding of *C. redtenbacheri* is to rear larvae on agaves in greenhouses in low larval densities and spaced irrigation conditions (Llanderal-Cázares et al., 2010). However, such an approach can be challenging if the goal is to efficiently mass-produce larvae. There is still very little known about how best to rear mezcal larvae and additional scientific research is needed to understand how captive insect breeding can become a central part of the agricultural industry in Mexico.

Many studies have used molecular diagnostics to examine food content (*e.g.*, Ghovvati et al., 2009; Lopez-Vizcón & Ortega, 2012; Pardo et al., 2018), as these tests allow for confirmation of proper product labeling. Studies like ours should continue to be conducted so that the foods we eat are frequently checked for accuracy.

##  Supplemental Information

10.7717/peerj.14948/supp-1 Supplemental Information 1 Voucher information for GenBank COI sequences that were downloaded and included in the present study

10.7717/peerj.14948/supp-2 Supplemental Information 2 Images of larvae that were examined in the present study

10.7717/peerj.14948/supp-3 Supplemental Information 3 All COI sequences included in the present study

10.7717/peerj.14948/supp-4 Supplemental Information 4 Pairwise distance (similarity) table for all samples in the COI data matrix

10.7717/peerj.14948/supp-5 Supplemental Information 5 Tree file for the tree shown in Fig. 4

## Additional Information and Declarations

### DNA Deposition

The following information was supplied regarding the deposition of DNA sequences: COI sequences are available at GenBank: OP654752 to OP654761. Sequences are also available in the Supplemental Files.

### Data Availability

The following information was supplied regarding data availability: Raw data are available in the Supplemental Files.
