# Supplementary figures and images for: Mezcal worm in a bottle: DNA evidence suggests a single moth species
Source: PeerJ. 2023 Mar 8;11:e14948. doi: 10.7717/peerj.14948 (PMC10007961; doi:10.7717/peerj.14948)

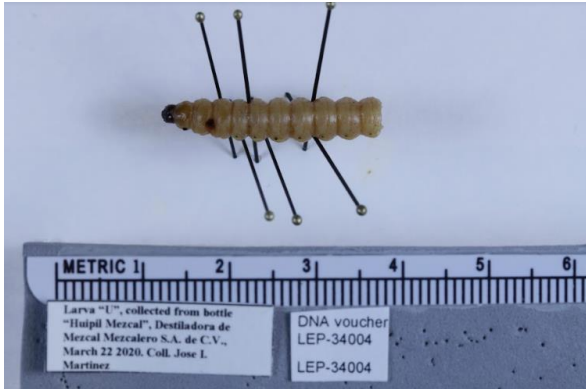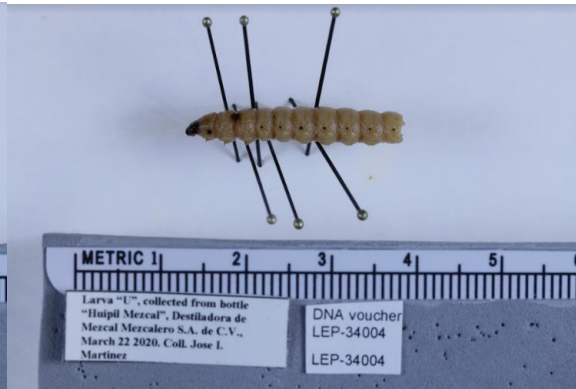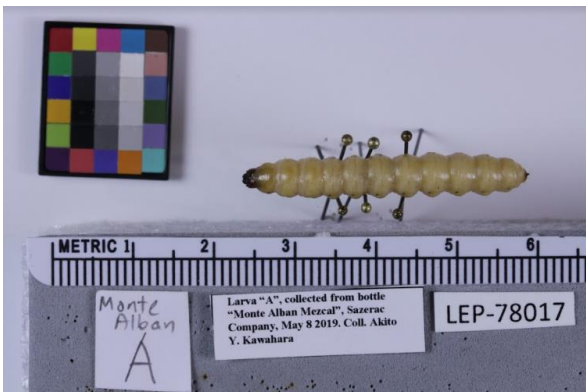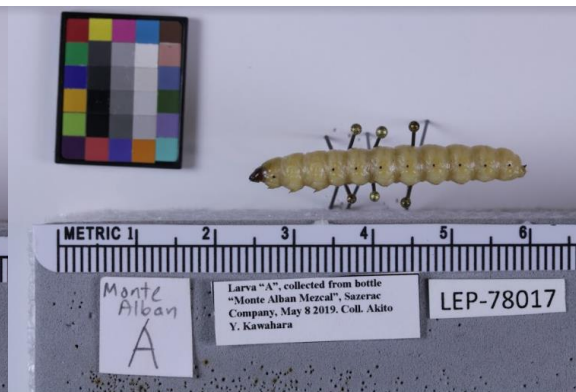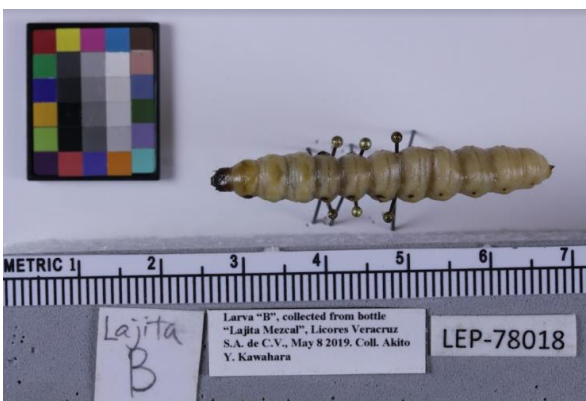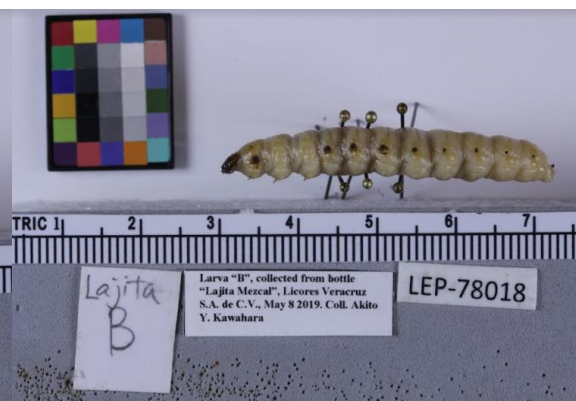

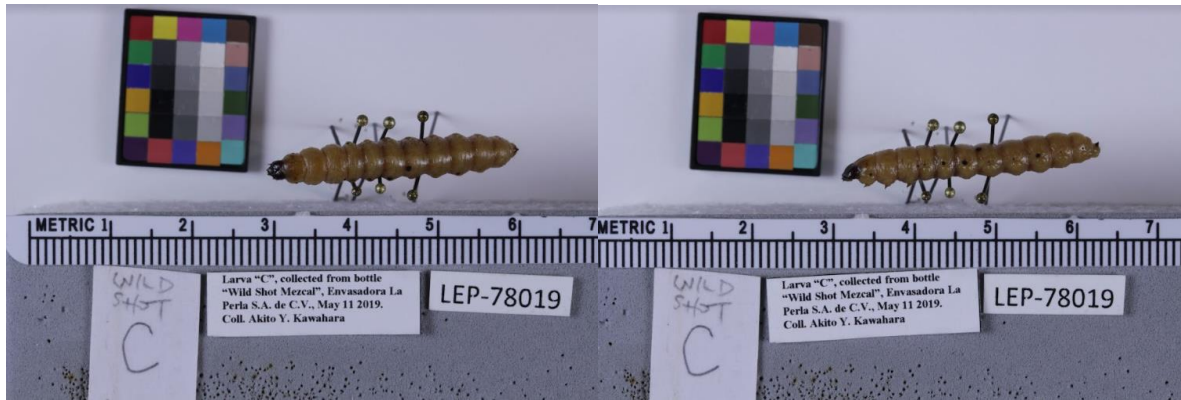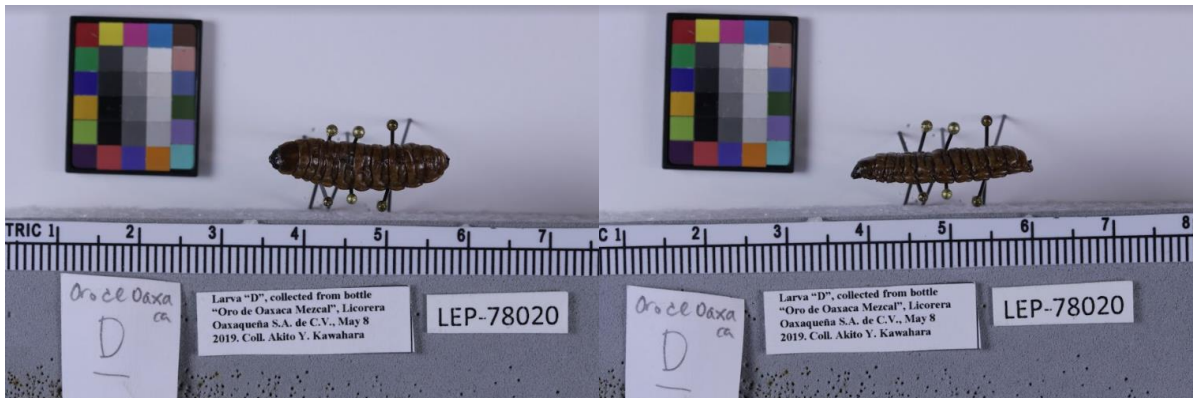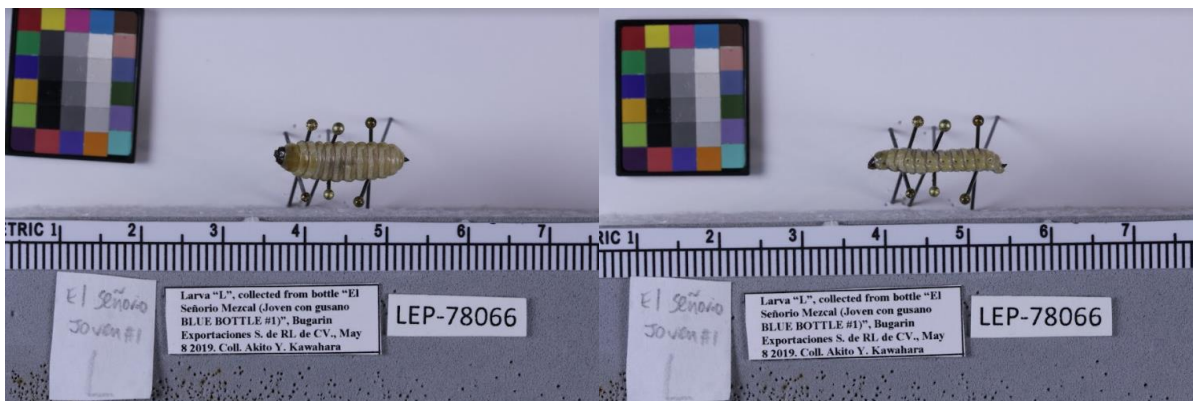

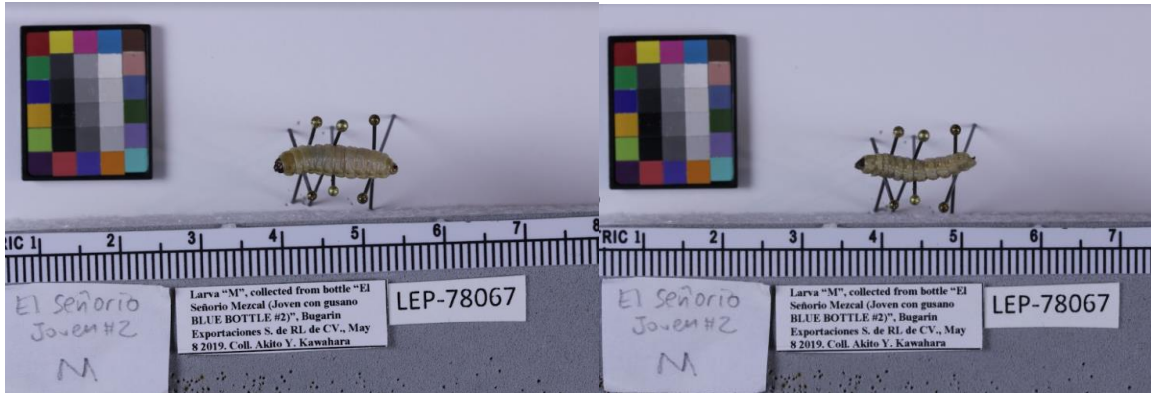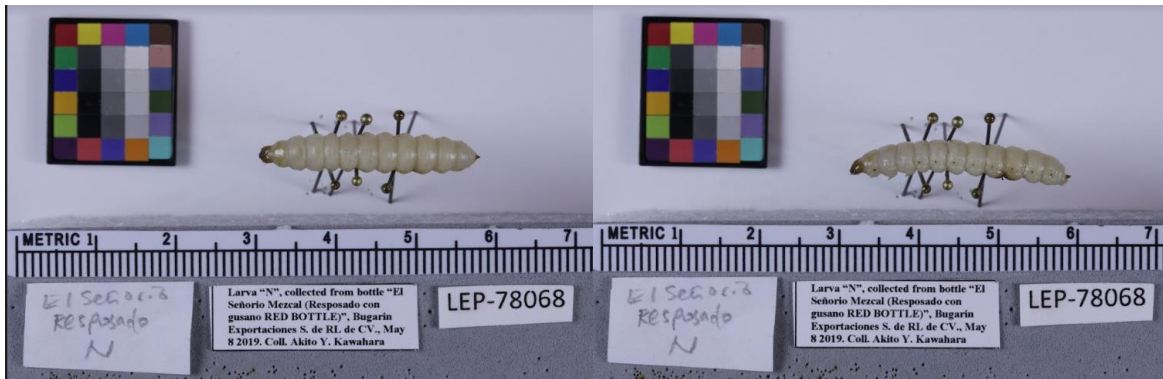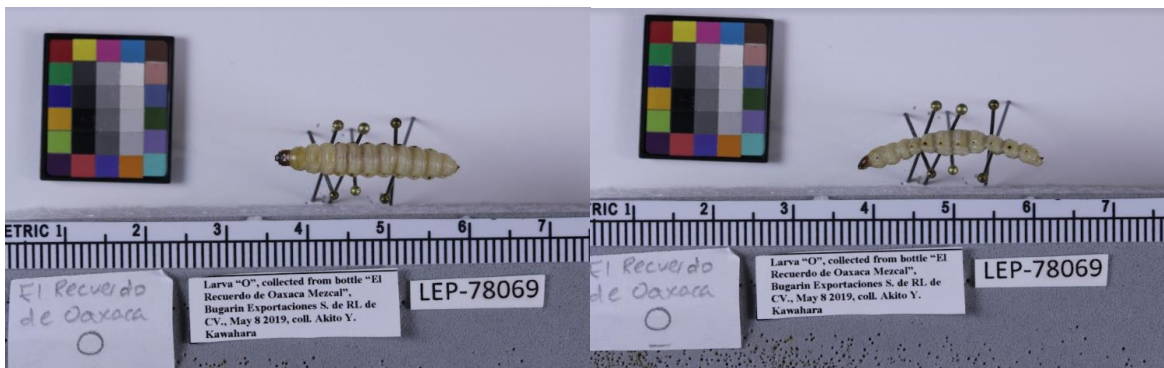

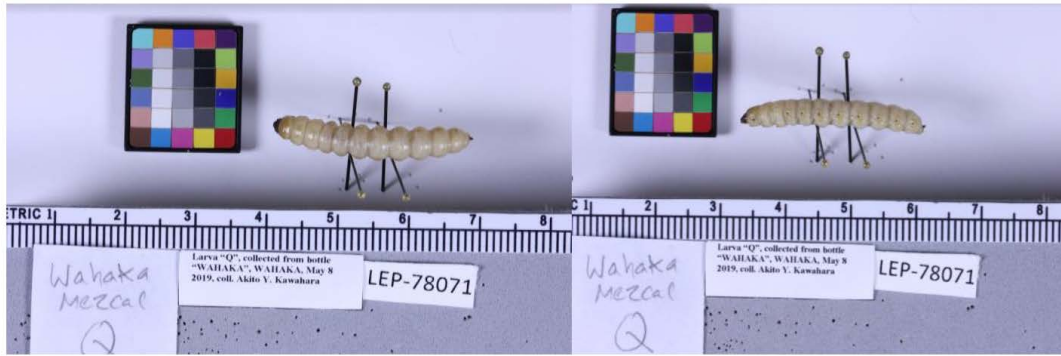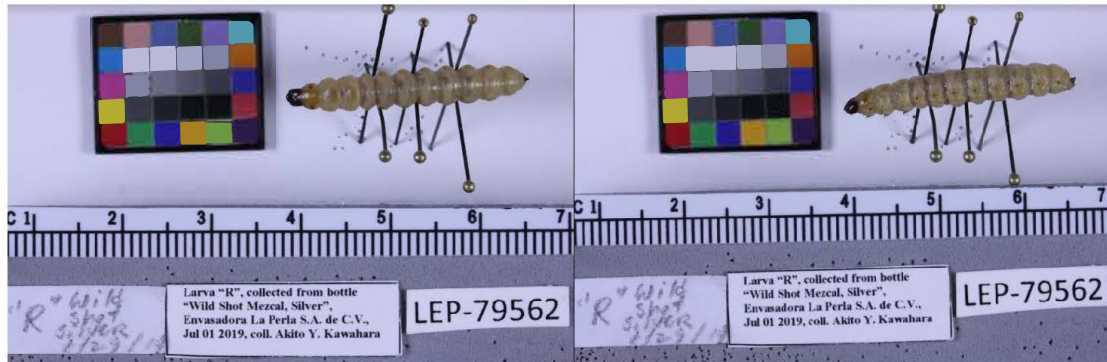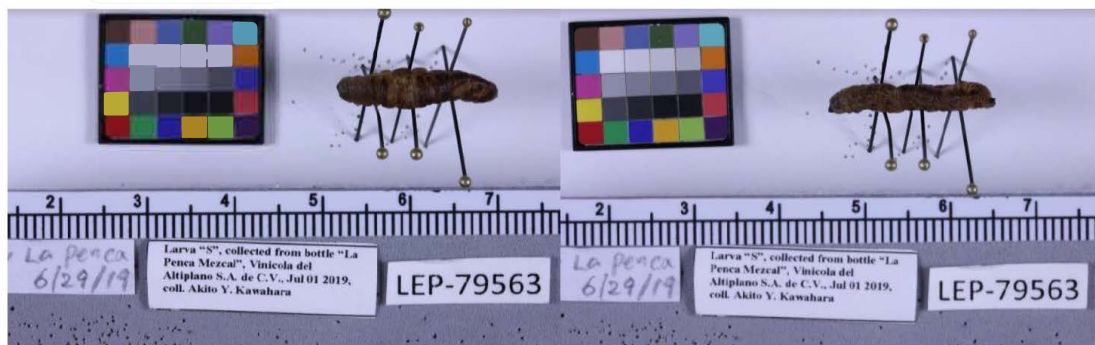

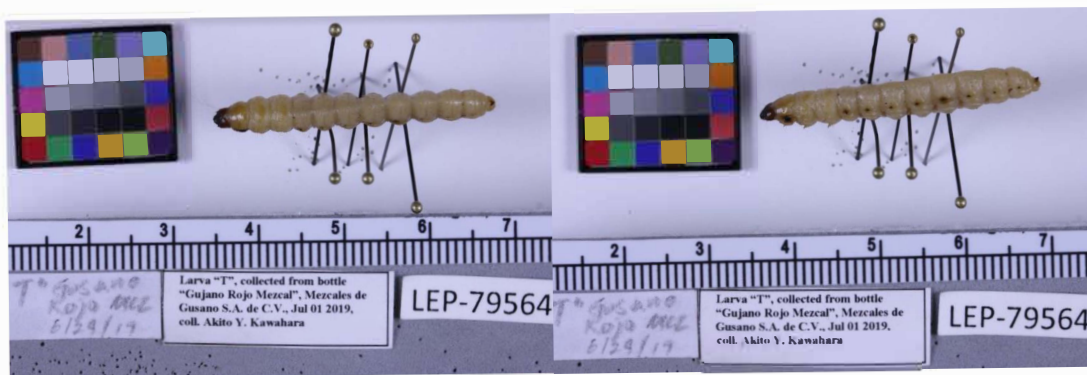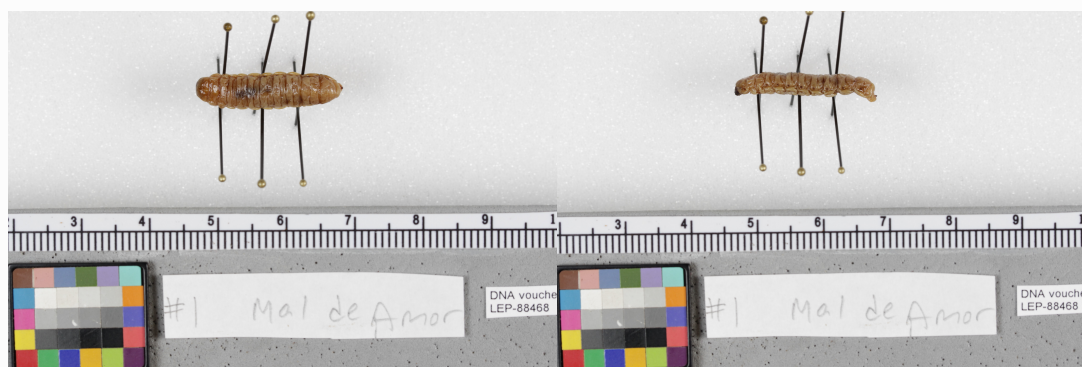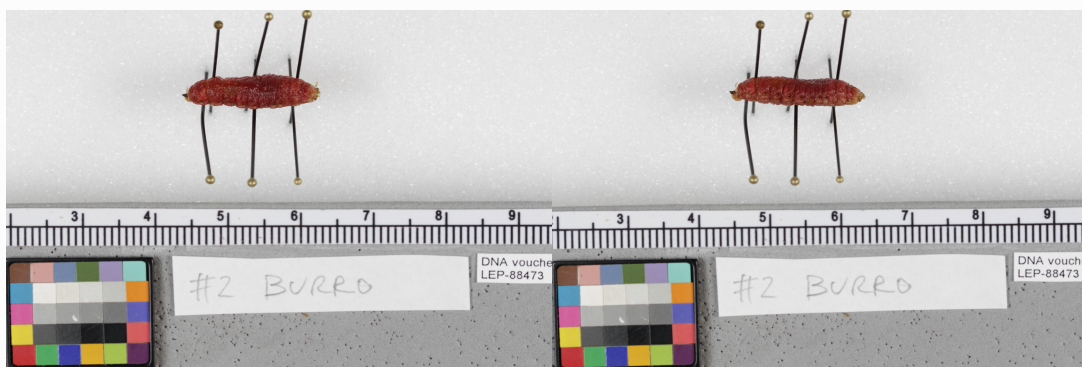

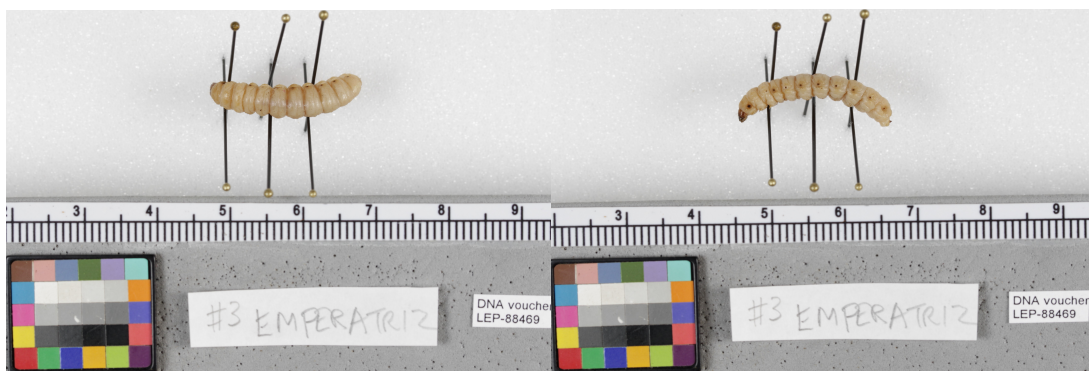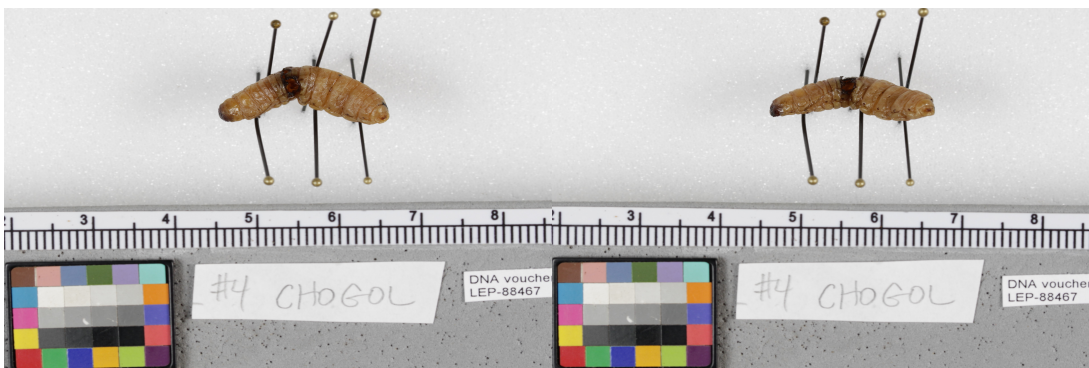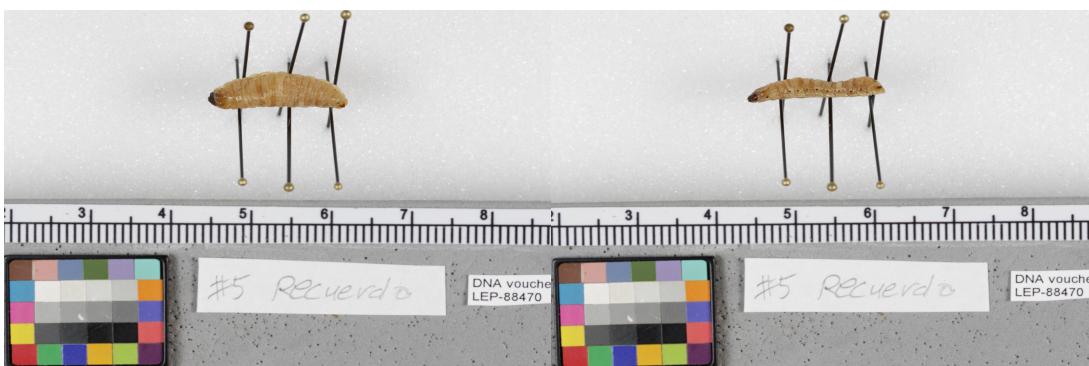

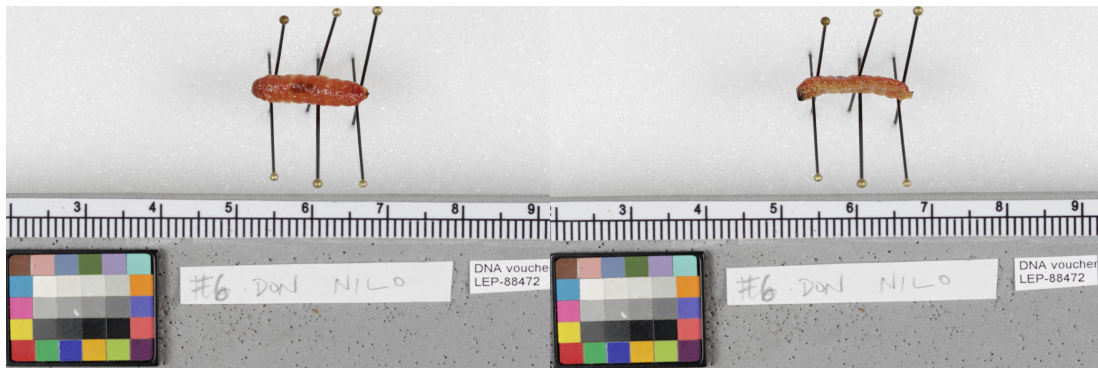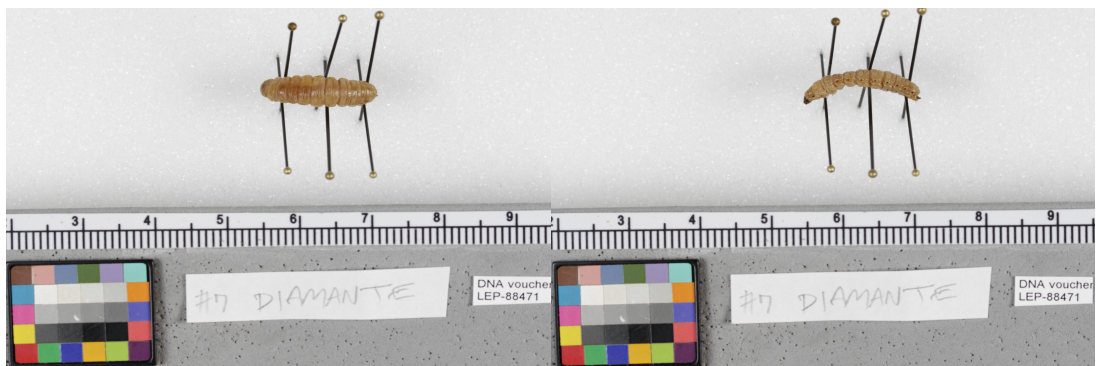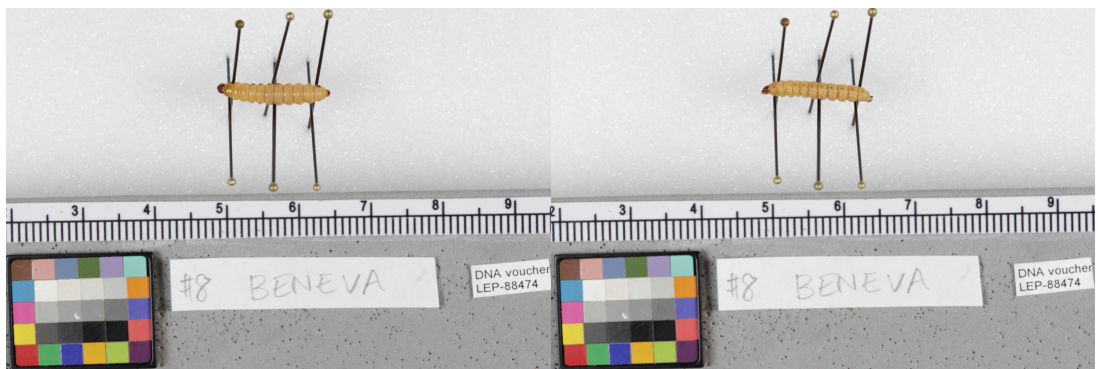

Supplement: Supplemental Information 2 [file peerj-11-14948-s002.pdf]
